# A Near-Infrared Ratiometric Fluorescent Probe for Highly Selective Recognition and Bioimaging of Cysteine

**DOI:** 10.3389/fchem.2019.00032

**Published:** 2019-02-01

**Authors:** Xuan Zhang, Li Zhang, Wei-Wei Ma, Yong Zhou, Zhen-Ni Lu, Suying Xu

**Affiliations:** ^1^Key Laboratory of Science and Technology of Eco-Textiles, Ministry of Education, College of Chemistry, Chemical Engineering & Biotechnology, Donghua University, Shanghai, China; ^2^State Key Laboratory of Fine Chemicals, Dalian University of Technology, Dalian, China; ^3^Department of Biochemistry, Faculty of Science, Beijing University of Chemical Technology, Beijing, China

**Keywords:** NIR ratiometric fluorescent probe, cysteine, benzothiazole derivative, living cells imaging, intramolecular charge transfer

## Abstract

A benzothiazole-based near-infrared (NIR) ratiometric fluorescent probe (**HBT-Cys**) was developed for discriminating cysteine (Cys) from homocysteine (Hcy) and glutathione (GSH). The probe was designed by masking phenol group in the conjugated benzothiazole derivative with methacrylate group that could be selectively removed by Cys, and therefore an intramolecular charge transfer (ICT) fluorescence was switched on in the NIR region. In the absence of Cys, the probe exhibited a strong blue fluorescence emission at 431 nm, whereas a NIR fluorescence emission at 710 nm was significantly enhanced accompanied by a decrease of emission at 431 nm in the presence of Cys, allowing a ratiometric fluorescence detection of Cys. The fluorescence intensity ratio (I_710nm_/I_431nm_) showed a good linear relationship with Cys concentration of 1–40 μM with the detection limit of 0.5 μM. The sensing mechanism was explored based on MS experimental analysis and DFT theoretical calculation. Moreover, the fluorescent probe was successfully used for fluorescence bioimaging of Cys in living cells.

## Introduction

It has been known that small molecular biothiols, such as L-cystein (Cys), homocysteine (Hcy), and glutathione (GSH) played vital roles in the maintenance of redox homeostasis, intracellular signal transduction, and human metabolism (Shahrokhian, 2001; Giles et al., 2003). Cys is a metabolic product of Hcy and a precursor of the antioxidant GSH, and its normal intracellular level remains to be 30–200 μM (Liu et al., 2014). The deficiency of Cys could cause edema, leucocyte loss, liver damage as well as neurotoxicity, whereas the excess levels of Cys might relate to cardiovascular and Alzheimer's diseases (Lipton et al., 2002; Shao et al., 2012; Dorszewska et al., 2016; Qi et al., 2018). Hence, it has attracted intense interest in the development of novel strategy for detection and imaging of the intracellular Cys, which will further contribute to the better understanding the pathology of associated diseases and their early diagnosis and treatment.

Design and synthesis of small molecule-based fluorescent probe received much attention in molecule recognition and fluorescence signaling in living biosystems due to many advantages of fluorescence technique, such as high sensitivity and selectivity, simplicity, *in vivo* bioimaging (De Silva et al., 1997; Chen et al., 2010, 2016; Chan et al., 2012; Yang et al., 2013; Li et al., 2014; Niu et al., 2015). For bioimaging application, the development of fluorescent probes with near-infrared (NIR, 650–900 nm) emission is more promising due to the merits of deeper tissue penetration and minimum interference from the indigenous fluorescence background of biosystem (Escobedo et al., 2010; Nolting et al., 2011; Yuan et al., 2013; Guo et al., 2014). Additionally, the ratiometric fluorescent probes could provide an inherent reliability originating from its effective self-calibration advantage by monitoring two well-resolved emissions (Kikuchi et al., 2004; Demchenko, 2010; Lee et al., 2015). Although a number of fluorescent probes for Cys detection and imaging have been reported, most of them could not discriminate Cys from Hcy/GSH due to their similar molecular structures and reactivity (Chen et al., 2017, 2018; Li et al., 2017; Liu X. et al., 2017; Nawimanage et al., 2017; Wang F. et al., 2017; Wang Q. et al., 2017, 2018a; Wu et al., 2017; Yin et al., 2017; Yue et al., 2017a,b; Zhang et al., 2017, 2018a,b; Hou et al., 2018; Kim et al., 2018; Ren et al., 2018; Song et al., 2018; Tian et al., 2018; Wang et al., 2018b; Wang J. et al., 2018; Wang L. et al., 2018; Sheng et al., 2019). Several ratiometric fluorescent probes for Cys have been developed (Lv et al., 2014; Feng et al., 2017; Liu G. et al., 2017; Wang F. et al., 2017; Wang L. et al., 2018 Wu et al., 2018; Yue et al., 2018; Zhu et al., 2018), but only a few of them showed the fluorescence emission in NIR region (Feng et al., 2017; Zhu et al., 2018). Therefore, the development of NIR ratiometric fluorescent probe for selective detection of Cys is still a challenging task.

**Scheme 1:**
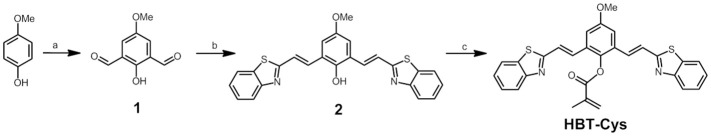
Synthesis of **1**, **2**, and **HBT-Cys**. Reagents and conditions: **(a)** hexamethylenetetramine, TFA, 110°C, 72 h; **(b)** 2-methylbenzothiazole, AcO_2_, 145°C, 56 h; pyridine, 115°C, 2 h; water, 100°C, 6 h; and **(c)** methacryloyl chloride, acetone, K_2_CO_3_, r.t., 8 h.

Recently, we have found that the π-conjugation extended benzothiazole derivatives exhibited short-wavelength fluorescence emission in non-polar solvent but the NIR emission in polar solvent, where the NIR fluorescence originated from the deprotonation of phenol group switching on an intramolecular charge transfer (ICT) process (Zhang et al., 2016b). In this work, we envisaged that the masking of phenol group in conjugated benzothiazole derivatives with methacrylate moiety, a Cys-selective recognition site, will result in a short-wavelength emission but a NIR fluorescence will appear in the presence of Cys due to the Cys-selectively induced deprotection, and therefore will allow a NIR ratiometric fluorescence detection of Cys. Accordingly, a new conjugated benzothiazole derivative (**HBT-Cys**, Scheme 1) was synthesized and developed as a novel NIR ratiometric fluorescent probe for Cys detection. The probe distinguished Cys well from GSH/Hcy in a ratiometric manner in aqueous solution and successfully applied in living cells imaging.

## Materials and Methods

All the chemicals are analytical grade that was used without purification and purchased from Sinopharm Chemical Reagents Corp. (Shanghai, China). Phosphate buffered saline (PBS, pH = 7.4) was prepared from K_2_HPO_4_ (0.1 M) and KH_2_PO_4_ (0.1 M). The stock solution of the probe **3** was prepared in DMF and all of others species solutions were prepared in deionized water. ^1^H NMR and ^13^C NMR spectra were recorded on a Bruker AVANCE III 400 MHz spectrometer. Mass spectra were obtained on AB Sciex MALDI-TOF/TOF™ MS. Fluorescence spectra were measured on Edinburgh FS5 spectrofluorometer with Ex/Em slit widths of 5 nm. The absolute fluorescence quantum yields were obtained on Edinburgh FS5 spectrofluorometer equipped with an integrating sphere (EI-FS5-SC-30). Absorption spectra were obtained on a SHIMADZU UV-1800 spectrophotometer. Confocal fluorescence imaging experiments in living Hela cells were carried out with a Carl Zeiss LSM 700 microscope. Theoretical calculations were performed based on the Gaussian 09 package (Frisch et al., 2010). The ground state and the first singlet excited state geometries of the compounds were optimized in the gas phase using density functional theory (DFT) and time-dependent density functional theory (TDDFT) at the B3LYP/6-31+G(d) level, respectively. The fluorescence emission properties were calculated using TDDFT based on the optimized first singlet excited state geometries, respectively.

HeLa cells were cultured in Dulbecco's Modified Eagle Medium (DMEM) supplemented with 10% fetal bovine serum at 37°C in a 95% humidity atmosphere under 5% CO_2_ environment. Then the cells were seeded in confocal microscope culture dishes with a density of 2 × 10^5^ cells per well. The cells were then incubated with probe **HBT-Cys** (20 μ M) for 120 min at 37°C, washed with PBS buffer (10 mM) three times to remove free probe. In the control experiments, the cells were pretreated with NEM (1 mM) for 30 min at 37°C, followed by washing with PBS for three times, and incubated with probe **HBT-Cys** (20 μM) for 120 min at 37°C. In another control experiment, the cells were pretreated with NEM (1 mM) for 30 min at 37°C, followed by washing with PBS three times, then incubated with Cys (200 μM) for 30 min, and further incubated with probe **HBT-Cys** (20 μ M) for 120 min at 37°C, respectively. All the cells were washed with PBS three times to remove free probe and then imaged at blue and red channels, respectively in a Carl Zeiss LSM 700 microscope.

The synthesis procedures are illustrated in Scheme 1. The compound **1** was facilely synthesized according to the similar procedure described previously (Zhang and Liu, 2016a). Briefly, under an N_2_ atmosphere, 4-methoxylphenol (20 mmol) and hexamethylenetetramine (60 mmol) were dissolved in TFA (15 mL) and refluxed at 110°C for 72 h. The mixture was then cooled down to room temperature and poured into a 3 M HCl solution (120 mL). The crude product was obtained by filtration and further purified by column chromatography (silica gel, CH_2_Cl_2_) to give **1** as a yellow solid (Figures S3, S4). Yield: 19%. ^1^H NMR (400 MHz, DMSO-d_6_), δ (ppm): 11.09 (s, 1H), 10.24 (s, 2H), 7.60 (s, 2H), 3.82 (s, 3H). ^13^C NMR (100 MHz, DMSO-d_6_), δ (ppm): 55.89, 121.34, 124.31, 152.12, 156.45, 191.70.

Under an N_2_ atmosphere, compound **1** (4 mmol) and 2-methylbenzothiazole (16 mmol) were refluxed in acetic anhydride (3 mL) at 145°C for 56 h. After cooling to room temperature, the solid was obtained by filtration and thoroughly washed by water. The obtained solid was further dissolved in pyridine (19 mL) and refluxed at 115°C for 2 h, then water (10 mL) was added and stirred at 100°C for another 6 h. After cooling down to room temperature, the crude product was collected by filtration and thoroughly washed by CH_2_Cl_2_ to give **2** as a yellow solid (Figures S5, S6). Yield: 72%. ^1^H NMR (400 MHz, DMSO-d_6_), δ (ppm): 9.55 (s, 1H), 8.12 (d, J = 4 Hz, 2H), 8.02-7.98 (m, 4H), 7.68 (d, J = 16 Hz, 2H), 7.53 (t, J = 8 Hz, 2H), 7.45 (t, J = 8 Hz, 4H), 3.86 (s, 3H); ^13^C NMR (100 MHz, DMSO-d_6_), δ (ppm): 55.60, 113.19, 122.12, 122.19, 122.42, 125.32, 125.90, 126.44, 132.05, 133.93, 147.90, 153.12, 153.40, 166.72. MALDI-TOF-MS: m/z calcd for C_25_H_18_N_2_O_2_S_2_, 442.08; found 442.8298 [M + H]^+^ (Figure S9).

Compound **2** (0.1 mmol) was dissolved in acetone (200 mL) and methacryloyl chloride (0.13 mmol in 5 mL acetone) was slowly added dropwise under stirring at 0°C in the presence of K_2_CO_3_ (2.0 mmol). Then the mixture was warmed to room temperature and stirred for another 8 h. The reaction mixture was filtrated and the filtrate was concentrated in vacuum. The residue was dissolved in CH_2_Cl_2_ and then washed with water and the organic layer was dried with MgSO_4_. After removing MgSO_4_ by filtration, the crude product was obtained by evaporation under reduced pressure and then purified by column chromatography (silica gel, CH_2_Cl_2_/ethyl acetate = 30:1 v/v) to give **HBT-Cys** as yellow solid (Figures S7, S8). Yield: 45%. ^1^H NMR (400 MHz, CDCl_3_), δ (ppm): 8.03 (d, J = 8 Hz, 2H), 7.86 (d, J = 8 Hz, 2H), 7.52-7.46 (m, 6H), 7.41 (t, J = 8 Hz, 2H), 7.28 (s, 2H), 6.61 (s, 1H), 5.99 (s, 1H), 3.92 (s, 1H), 2.20 (s, 3H). ^13^C NMR (100 MHz, CDCl_3_), δ (ppm): 18.67, 55.81, 112.86, 121.59, 123.13, 124.90, 125.86, 126.61, 128.52, 130.24, 130.99, 134.14, 135.07, 141.26, 153.31, 157.64, 165.86, 166.61. MALDI-TOF-MS: m/z calcd for C_29_H_22_N_2_O_3_S_2_, 510.11; found 511.2485 [M + H]^+^, 533.2472 [M + Na]^+^ (Figure S10).

## Results and Discussions

### Design and Synthesis

To design a selective fluorescent probe for Cys, a methacrylate group was chosen as the recognition site which can specifically react with Cys *via* conjugate addition/cyclization reaction (Yang et al., 2011; Ma et al., 2017). The conjugated benzothiazole derivatives displayed the large Stokes' shifted NIR fluorescence emission in deprontonated phenolate anion form due to the strong electron-donating ability of phenolate anion induced an occurrence of ICT (Karton-Lifshin et al., 2012; Zhang et al., 2016b), but showed a short-wavelength fluorescence in the neutral phenol form, which allowed a ratiometric detection manner based on two emissions. The novel fluorescent probe **HBT-Cys** was thus readily synthesized by combining methacrylate group into conjugated benzothiazole derivative. As shown in Scheme 1, the 2,6-diformyl-4-methoxylphenol (**1**) was firstly synthesized from commercially available 4-methoxylphenol *via* Duff reaction, then the conjugated benzothiazole derivative (**2**) was obtained by a direct condensation reaction between **1** and commercially available 2-methylbenzothiazole, and finally the probe **HBT-Cys** was easily afforded by treating **2** with methacryloyl chloride in acetone. The chemical structures of the probe **HBT-Cys** and intermediate compounds were characterized by ^1^H NMR, ^13^C NMR, and MALDI-TOF-MS.

### Spectral Properties

**Figure 1:**
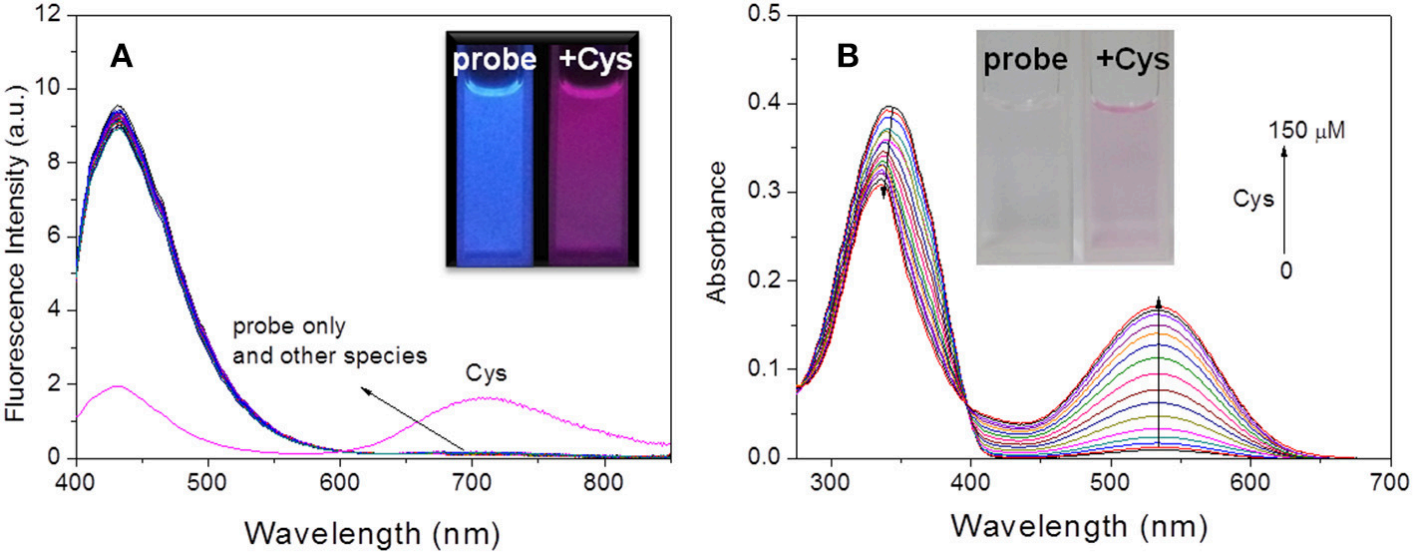
Fluorescence spectra of **HBT-Cys** (10 μM) in the absence and presence of 15 equiv. of various amino acids and biologically relevant species **(A)**. Absorption spectra of **HBT-Cys** (10 μM) upon addition of 0–150 μM Cys **(B)**. Insets are the fluorescence **(A)** and solution **(B)** color change of the probe in the absence and presence of Cys, where the fluorescence in **(A)** was obtained by exposing the solution under UV lamp at 365-nm. The measurements were performed in aqueous PBS buffer solution (pH = 7.4, 50% DMF) and the excitation wavelength was 396 nm for fluorescence measurement.

The conjugated benzothiazole derivative **2** showed a short-wavelength emission at 500 nm in CHCl_3_ but a NIR emission at 730 nm in DMF with fluorescence quantum yield of 0.36 and 0.24, respectively (Figure S1). The NIR fluorescence emission was observed at 710 nm with a fluorescence quantum yield of 0.085 even in the PBS buffer solution (pH = 7.4, containing 50% DMF). These excellent fluorescence properties allowed compound **2** as a suitable platform to construct the fluorescent probe. As expected, the probe **HBT-Cys** showed a short-wavelength emission at 431 nm in the PBS buffer solution (pH = 7.4, containing 50% DMF), but a NIR fluorescence located at 710 nm appeared with decrease of the short-wavelength emission in the presence of Cys (Figure 1A). In contrast, the fluorescence spectra of the probe **HBT-Cys** showed no distinct change upon addition of others species such as common amino acids (GSH, Cys, Hcy, Asp, Asn, Ser, Pro, Ala, Gly, Val, Leu, lle, Thr, Arg, Glu, Gln, Tyr, His, Met, Phe, Trp, Lys, Tau), cations (Na^+^, K^+^, Ca^2+^, Mg^2+^), anions (${\text{SO}}_{4}^{2-}$, ${\text{NO}}_{3}^{-}$, Cl^−^), Na_2_S, mercaptoacetic acid and glucose. An obvious fluorescence color change from blue to red was observed in the presence of Cys (Figure 1A). This indicates that the probe **HBT-Cys** exhibited a high selectivity toward Cys in aqueous solution. Meanwhile, the probe **HBT-Cys** only displayed a strong absorption at 343 nm in the PBS buffer solution (pH = 7.4, containing 50% DMF), and the peak at 343 nm gradually decreased with a significant increase of the absorption at 533 nm upon addition of various amounts of Cys, where an isosbestic point was observed at 396 nm and the solution color turned pink from colorless (Figure 1B). No significant absorption spectral change was observed in the presence of others species. These spectral properties suggested that the **HBT-Cys** could serve as a NIR ratiometric fluorescent probe for high selective detection of Cys over Hcy/GSH.

### Sensing Mechanism

**Scheme 2:**
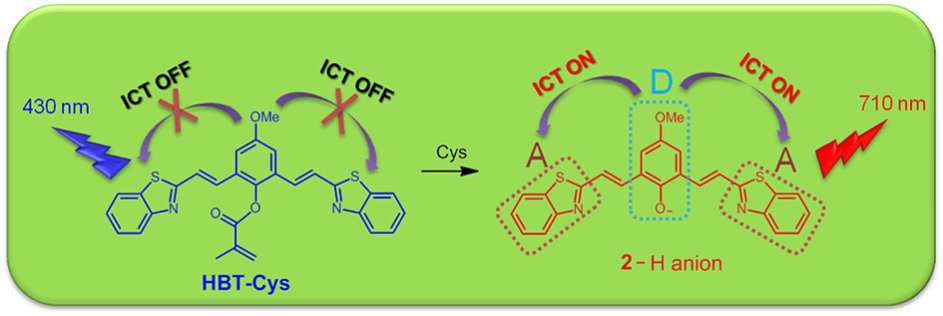
The proposed sensing mechanism of the probe **HBT-Cys** toward Cys.

**Figure 2:**
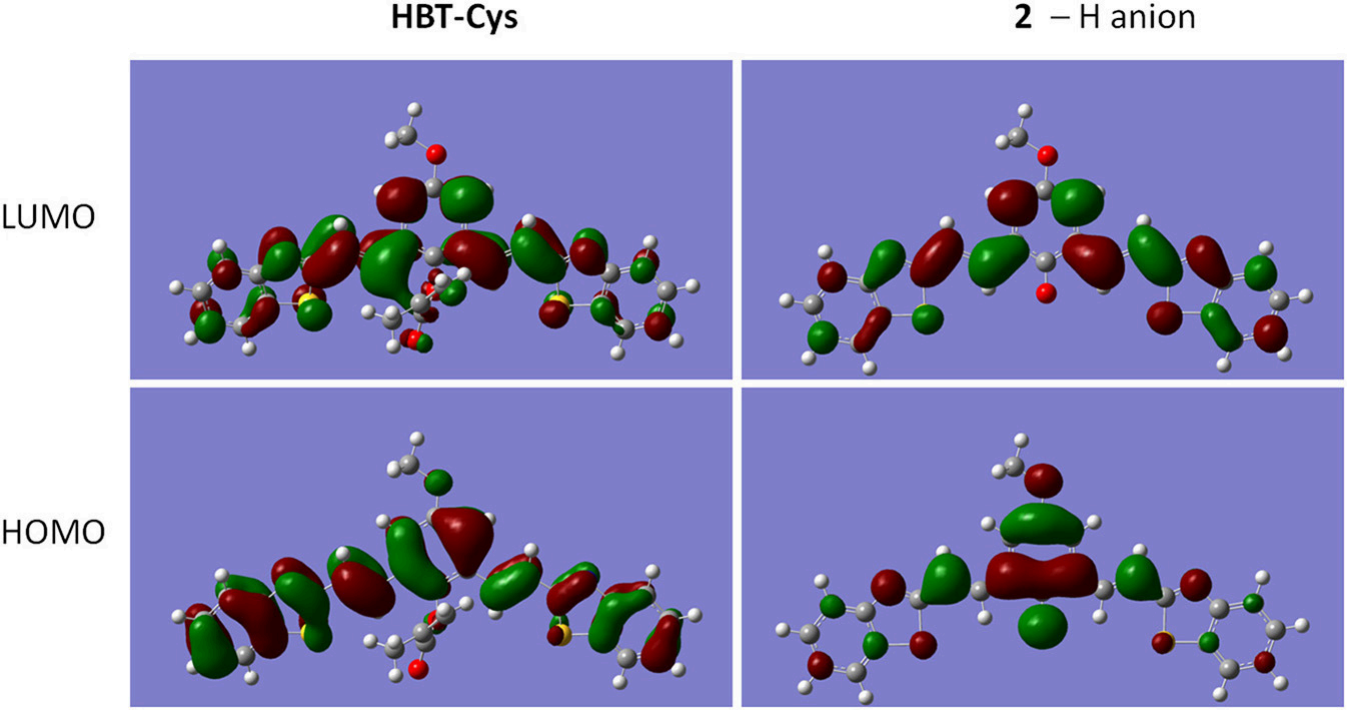
LUMO and HOMO orbitals of **HBT-Cys** and **2**–H anion in the ground state.

To confirm that probe **HBT-Cys** has been transformed into **2** in the presence of Cys as shown in Scheme 2, ESI-MS mass analysis of a mixture solution of the probe **HBT-Cys** (10 μM) with 10 equiv. Cys was conducted. A prominent peak at m/z = 441 corresponding to the [**2**–H]^−^ anion was observed (Figure S2), suggesting the fact that Cys took the methacrylate moiety away from probe **HBT-Cys**. The DFT calculation was further performed to gain better insights into the NIR fluorescence and signaling mechanism. Figure 2 presented the optimized ground state structures of both the probe **HBT-Cys** and **2**–H anion. Obviously, the π electrons of the probe **HBT-Cys** were well-delocalized on the whole molecular skeleton on both the lowest unoccupied molecular orbital (LUMO) and the highest occupied molecular orbital (HOMO). When the π electrons of **2**–H anion were still delocalized on the whole molecular skeleton on the LUMO, they mainly localized on the phenolate group on the HOMO, implying a potential ICT process from the phenolate donor (D) to two benzothiazole acceptors (A) as shown in . It has been known that the phenol moiety is a latent electron donor but acts as a strong electron donor when it transformed into phenolate, and could therefore switch on an ICT emission (Karton-Lifshin et al., 2012; Zhang et al., 2016b). Based on the TD-DFT calculation on the excited state, the fluorescence emission wavelengths were predicted to be 739 nm for the **2**–H anion. The well-reproduction of the experimental result implied that the reliability of present theoretical calculation level. Thus, the signaling mechanism could be rationalized as Cys induced removal of methacrylate group and switch on of ICT emission, shown in Scheme 2.

### Response Time and pH Influence

**Figure 3:**
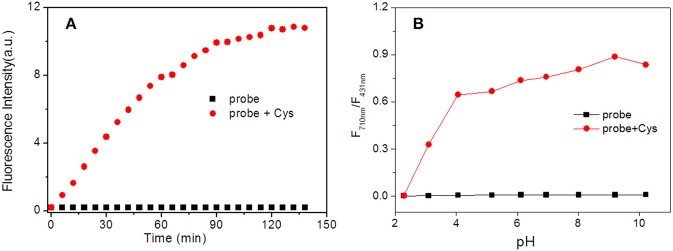
Time **(A)** and pH **(B)** effect on fluorescence intensity of **HBT-Cys** in the absence and presence of Cys. The fluorescence intensity measured at 710 nm in **(A)**. The measurements were performed in aqueous PBS buffer solution (pH = 7.4, 50% DMF) and the excitation wavelength was 396 nm for fluorescence measurement.

The time-dependent fluorescence response of the probe **HBT-Cys** with and without Cys was performed, respectively. As shown in Figure 3A, the NIR fluorescence intensity at 710 nm was dramatically increased over 60 min and leveled off after about 120 min in the presence of Cys. Without Cys, the NIR fluorescence of the probe **HBT-Cys** displayed no significant changes, suggesting that the probe itself is stable enough under experimental condition. Thus, the spectral measurements of **HBT-Cys** were carried out after 120 min upon addition of Cys in the solution.

The pH influence on the fluorescence of the probe both in the absence and presence of Cys were investigated. Without Cys, the fluorescence intensities ratio (I_710nm_/I_431nm_) displayed negligible changes over the range of pH 2.0–10.0 (Figure 3B). However, in the presence of Cys, the I_710nm_/I_431nm_ ratio showed a drastic enhancement after pH > 3 and remained to be almost constant over whole pH region examined. This revealed that the present probe could work in a broad pH region (pH 3.0–10.0) and suitable for imaging under physiological conditions.

### Ratiometric Fluorescence Detection and Imaging of Cys

**Figure 4:**
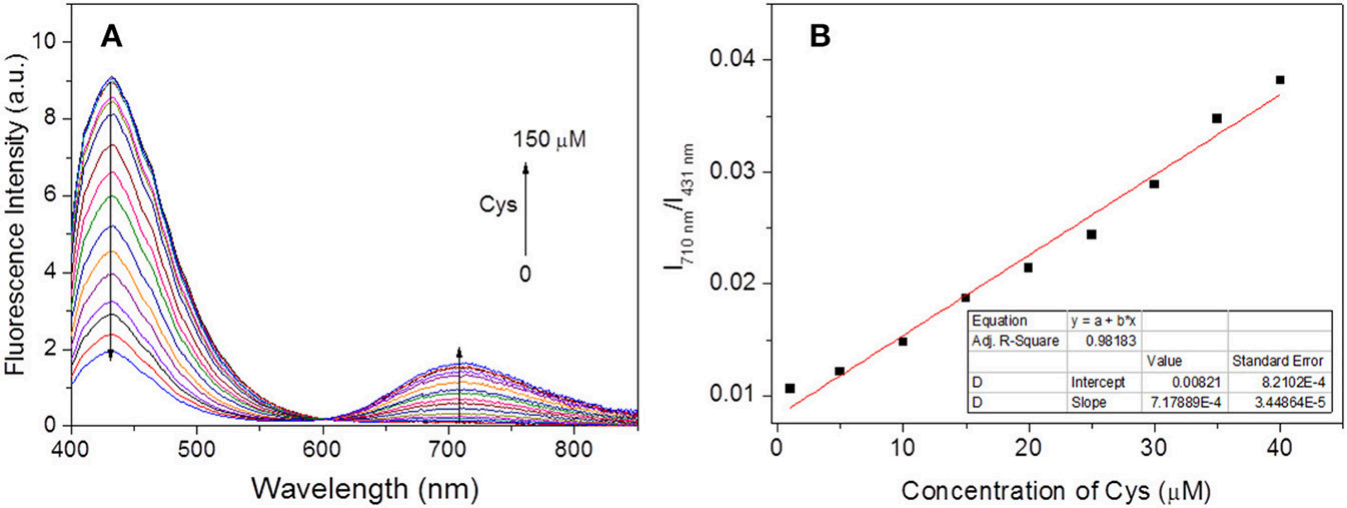
Evolution of fluorescence spectra of **HBT-Cys** (10 μM) with addition of various amount of Cys (0–150 μM) **(A)** and the corresponding linear relationship of fluorescence intensity ratio (I_710nm_ /I_431nm_) vs. concentration of Cys (0–40 μM) **(B)**. The measurements were performed in aqueous PBS buffer solution (pH = 7.4, 50% DMF) and the excitation wavelength is 396 nm for fluorescence measurement.

Under the optimal experimental conditions, the ratiometric fluorescence titrations toward Cys were performed. With excitation at 396 nm (an isosbestic point in absorption spectra, Figure 1B), it could be seen that the fluorescence emission gradually decreased at 431 nm and increased at 710 nm with increasing Cys amounts up to 150 μM (Figure 4A). The I_710nm_ /I_431nm_ ratios followed a good linear relationship (*R*^2^ = 0.9818) with Cys concentration ranging from 1–40 μM (Figure 4B). The detection limit was estimated to be 0.5 μM according to S/N = 3. Hence, the probe **HBT-Cys** could detect Cys quantitatively by ratiometric fluorescence method with excellent sensitivity.

**Figure 5:**
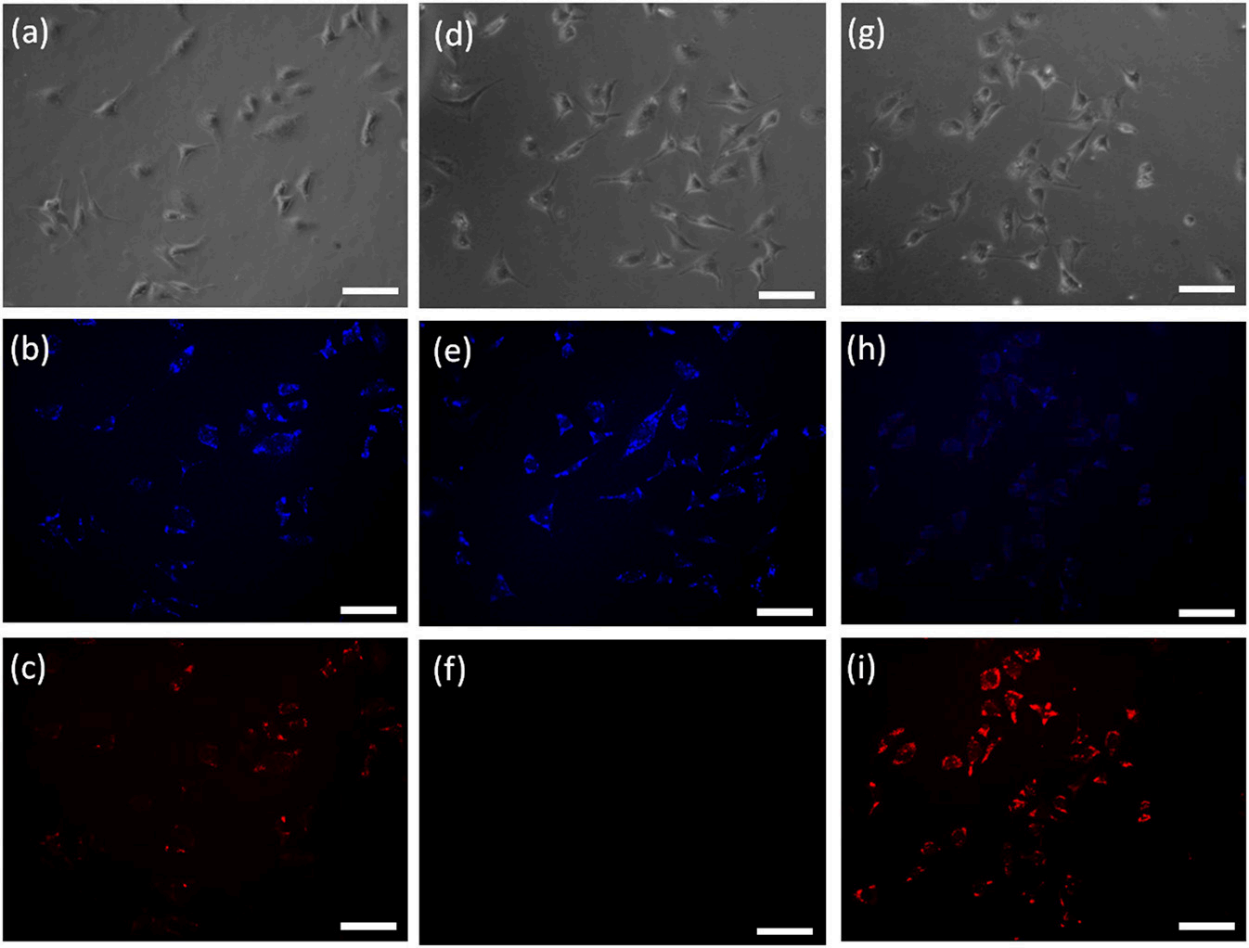
Confocal fluorescence images of living Hela cells incubated with the probe **HBT-Cys** (20 μM) for 120 min at 37°C: cells without treatment **(a–c)**, cells with pre-treatment of NEM (1 mM) **(d–f)**, and cells with pre-treatment of NEM (1 mM) and further addition of 200 μM Cys **(g–i)**. **(a,d,g)** are the bright-field images; **(b,c,e,f,h,i)** are the fluorescence images at blue and red channels, respectively. The scale bar represented 100 μm.

To evaluate the potential practical applications of probe **HBT-Cys**, the fluorescence imaging of Cys in living HeLa cells were also performed with a Carl Zeiss LSM 700 microscope, where dual blue and red channels were monitored, respectively (Figure 5). When HeLa cells were incubated with the probe **HBT-Cys** (20 μM) for 120 min at 37°C, both blue and red fluorescence emissions were observed in two channels (Figures 5b,c), where the NIR emission resulted from the intracellular Cys induced removal of methacrylate group in the probe. In the control experiment, HeLa cells were pretreated with N-ethylmaleimide (NEM, a known scavenger for Cys, 1 mM for 30 min), and thereafter incubated with probe **HBT-Cys** (20 μM) for another 120 min. While the blue fluorescence remained, there was no fluorescence in the red channel (Figures 5e,f). Then the NEM-pretreated HeLa cells were further sequentially incubated with Cys (200 μM) for 30 min, the probe **HBT-Cys** (20 μM) for 120 min at 37°C. As a result, a bright fluorescence in the red channel was again observed inside cells accompanied by a weak fluorescence in the blue channel (Figures 5h,i). These results suggested that the probe **HBT-Cys** can serve as a promising fluorescent probe for Cys imaging in living cells.

## Conclusions

In summary, a benzothiazole-based NIR ratiometric fluorescent probe **HBT-Cys** was developed for selective detection of Cys over Hcy and GSH in aqueous solution. The probe was designed by masking the phenol group in the conjugated benzothiazole derivative through methacrylate group that acts both as a trigger of the ICT fluorescence and recognition site for Cys. Upon addition of Cys, the NIR fluorescence emission at 710 nm was significantly increased with decrease of the fluorescence emission at 431 nm. The fluorescence intensity ratio (I_710nm_/I_431nm_) showed a linear relationship with Cys concentration of 1–40 μM with the detection limit of 0.5 μM. Based on mass analysis and DFT calculation, the signaling mechanism of Cys induced removal of methacrylate group and switch-on of the ICT fluorescence was proposed. The fluorescent probe was also successfully used for bioimaging of Cys in living cells, which would provide guidelines for design of novel ratiometric fluoresencent probes in future.

## Author Contributions

LZ and W-WM were responsible for designing and performing the experiments. YZ and Z-NL were responsible for the characterization of compounds. SX and XZ were responsible for drafting and discussing the manuscript.

### Conflict of Interest Statement

The authors declare that the research was conducted in the absence of any commercial or financial relationships that could be construed as a potential conflict of interest.
