# Supplementary material for: A Near-Infrared Ratiometric Fluorescent Probe for Highly Selective Recognition and Bioimaging of Cysteine
Source: Front Chem. 2019 Feb 1;7:32. doi: 10.3389/fchem.2019.00032 (PMC6367217; doi:10.3389/fchem.2019.00032)
Supplement: Supplementary file 1 [file Data_Sheet_1.docx]

Supplementary Material

A Near-Infrared Ratiometric Fluorescent Probe for Highly Selective Recognition and Bioimaging of Cysteine

Xuan Zhang^1,2^*, Li Zhang^1,#^, Wei-Wei Ma^1,#^, Yong Zhou^1^, Zhen-Ni Lu^1^ and Suying Xu^3^*

^1^ Key Laboratory of Science and Technology of Eco-Textiles, Ministry of Education, College of Chemistry, Chemical Engineering & Biotechnology, Donghua University, Shanghai 201620, China.

^2^ State Key Laboratory of Fine Chemicals, Dalian University of Technology, Dalian 116024, China.

^3^ Department of Biochemistry, Faculty of Science, Beijing University of Chemical Technology, Beijing 100029, China.

*** Correspondence:**Xuan Zhang and Suying Xu
xzhang@dhu.edu.cn; syxu@mail.buct.edu.cn

**^#^** These authors contributed equally to this work.

**Contents:**

**Figure S1** Fluorescence spectra of **2** in CHCl_3_, DMF and PBS solution (pH = 7.4) containing 50% DMF.

**Figure S2** ESI spectrum of **HBT-Cys** upon addition of 10 equiv of Cys.

**Figure S3** ^1^H NMR spectrum of **1**.

**Figure S4** ^13^C NMR spectrum of **1**.

**Figure S5** ^1^H NMR spectrum of **2**.

**Figure S6** ^13^C NMR spectrum of **2**.

**Figure S7** ^1^H NMR spectrum of **HBT-Cys**.

**Figure S8** ^13^C NMR spectrum of **HBT-Cys**.

**Figure S9** MALDI-TOF-MS spectrum of **2**.

**Figure S10** MALDI-TOF-MS spectrum of **HBT-Cys**.

## Supplementary Figures

**
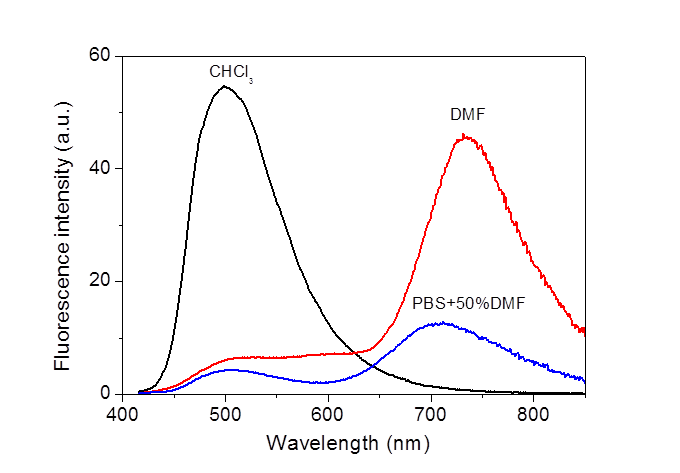
**

**Figure S1** Fluorescence spectra of **2** in CHCl_3_, DMF and PBS solution (pH = 7.4) containing 50% DMF.

**
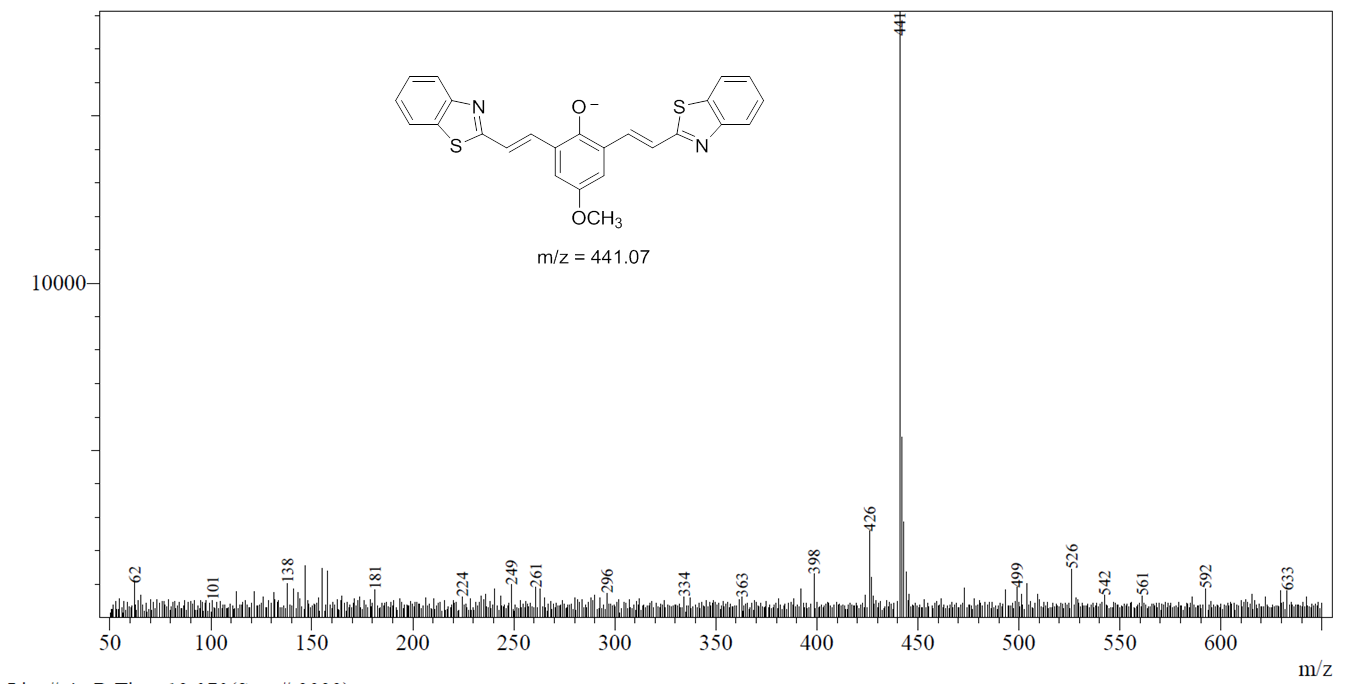
**

**Figure S2** ESI spectrum of **HBT-Cys** upon addition of 10 equiv of Cys.

**Figure S3** ^1^H NMR spectrum of **1**.

**Figure S4** ^13^C NMR spectrum of **1**.

**Figure S5** ^1^H NMR spectrum of **2**.

**Figure S6** ^13^C NMR spectrum of **2**.

**Figure S7** ^1^H NMR spectrum of **HBT-Cys**.

**Figure S8** ^13^C NMR spectrum of **HBT-Cys**.

**
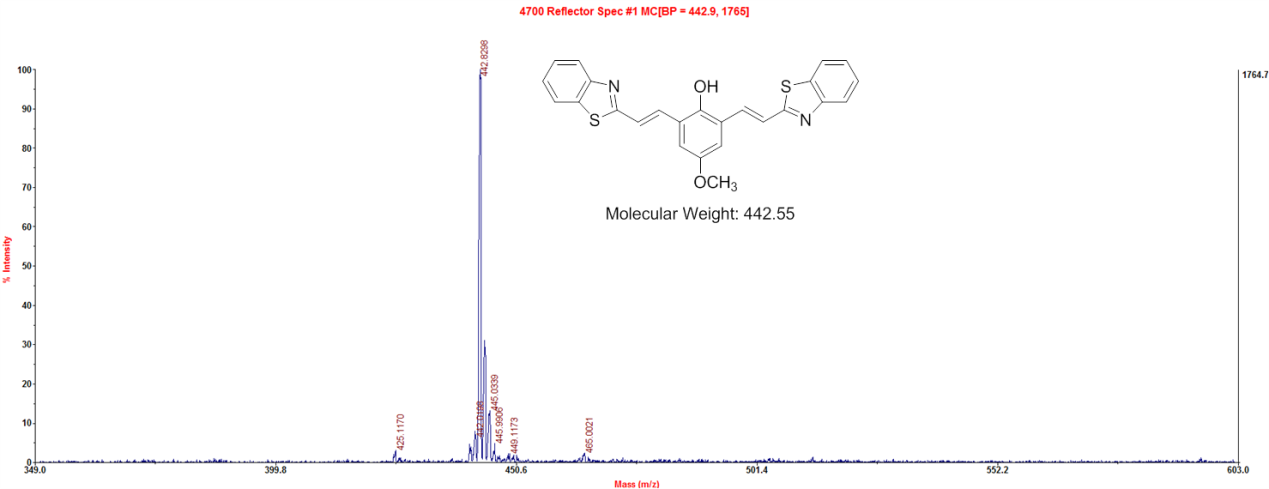
**

**Figure S9** MALDI-TOF-MS spectrum of **2**.

**
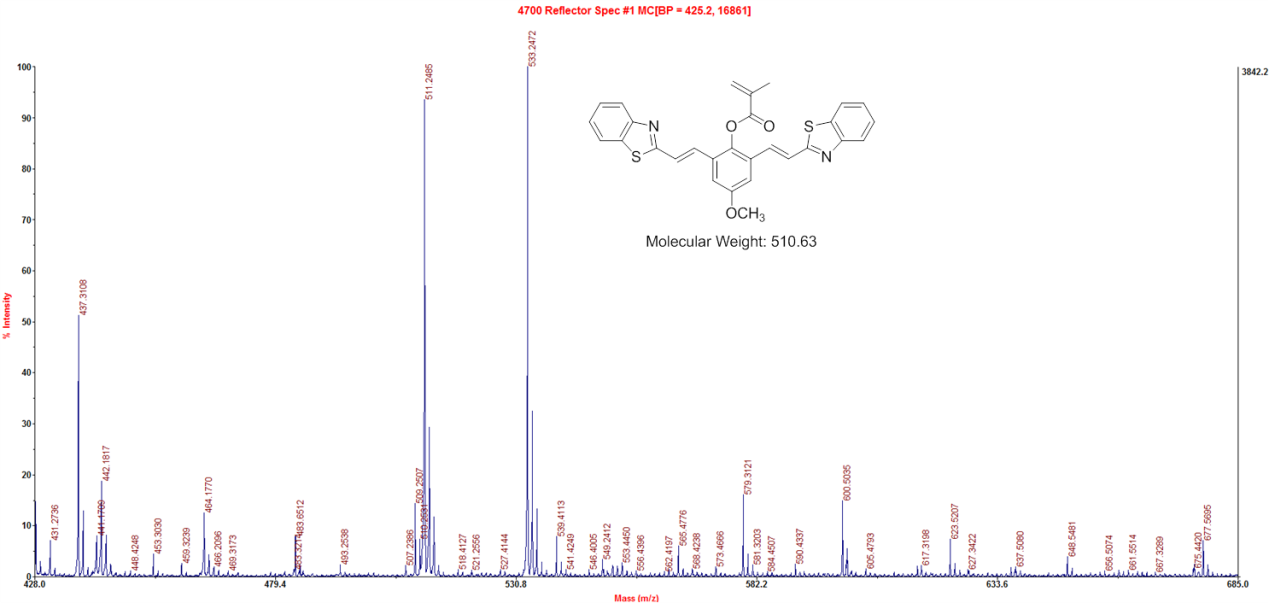
**

**Figure S10** MALDI-TOF-MS spectrum of **HBT-Cys**.
